# The Influence of Salt Sensitivity Phenotype on Sodium Excretion and Diuresis: A Chrononutrition Pilot Study

**DOI:** 10.1155/2022/9608962

**Published:** 2022-01-31

**Authors:** Upeksha Sewwandi Alwis, Irina Verbakel, Kim Pauwaert, Joris Delanghe, Lien Dossche, John Van Camp, Saskia Roggeman, Karel Everaert

**Affiliations:** ^1^Department of Human Structure and Repair, Ghent University, 9000 Ghent, Belgium; ^2^Department of Diagnostic Sciences, Ghent University, 9000 Ghent, Belgium; ^3^Department of Internal Medicine and Pediatrics, Ghent University, 9000 Ghent, Belgium; ^4^Department of Pediatric Nephrology, Ghent University, 9000 Ghent, Belgium; ^5^Department of Food Technology, Safety and Health, Ghent University, 9000 Ghent, Belgium; ^6^Research and Policy Department, Psychiatric Center Sint-Jan-Baptist, 9060 Zelzate, Belgium

## Abstract

**Background:**

Chrononutrition studies on interaction of diet/nutrients on endogenous circadian clocks and meal timing on metabolic homeostasis may be of importance in the management of nocturnal polyuria (NP), owing to loss of circadian rhythm in nighttime urination. Dietary salt restriction is an increasingly popular lifestyle recommendation for NP patients.

**Aim:**

This study aims to evaluate the effect of an acute salt load on diuresis and to study the phenomenon of salt sensitivity. *Methodology*. Young, healthy men (*n* = 21, fasted and sober) ingested 500 ml of water on the control day and 8 g and 12 g of salt with water (500 ml) on two other days. Blood and urine samples were collected at 0 hrs, 2 hrs, and 4 hrs and voided volumes were recorded. Diuresis, serum and urine osmolality, sodium, potassium, urea, and creatinine were measured. Salt sensitivity was determined based on the rate of sodium excretion.

**Results:**

Compared to 8 g, ingestion of 12 g of salt significantly increased diuresis after 4 hrs. Pure water load induced fast diuresis, whereas salt and water load initially reduced diuresis and promoted late increase in diuresis. The total voided volume was significantly lower in the salt sensitive individuals.

**Conclusion:**

Taken together, salt sensitivity profile and type and time of fluid intake are important considerations to build effective personalized lifestyle recommendations for NP patients, which needs further investigation.

## 1. Introduction

A wide range of biological and physiological functions in the body follow circadian rhythms, which are orchestrated by the circadian clocks present in many organs and cells, including brain, kidney, and bladder [1]. Over the last few years, a growing body of evidence has emerged on the interaction of diet/nutrients and meal timing on metabolism and endogenous circadian rhythm [2–4]. This area of “Chrononutrition” may also be of importance in the management of nocturia, in relation to loss of circadian rhythm in urine production [5]. Nocturia or the act of waking up at night to urinate during the main sleeping period [6] is a very common and bothersome lower urinary tract symptom (LUTS), and it is most common in the elderly [7]. Nighttime voiding in healthy humans is regulated through circadian rhythms, by decreasing brain arousal and kidney urine production rate, whilst increasing functional bladder capacity, which all three combined, prevents nocturnal micturition. In most patients suffering from nocturia, disturbances in this circadian rhythm are shown [1].

In an in vivo study, Gizowski et al. showed that an acute salt load could alter the circadian clock timing and could drive unscheduled homeostatic responses via clock-output networks [8]. On the other hand, excessive dietary salt intake has been identified as a risk factor for nocturia [9] and dietary salt reduction significantly improved nocturia in patients with high salt intake. Thus, the effect of high salt intake in nocturia patients has garnered a great attention in the management of nocturia [10–12]. However, individuals respond differently to an excess dietary salt intake, and this variability has been extensively studied in the phenomenon of salt sensitivity of blood pressure (BP) [13]. Salt sensitive BP has been explained by various mechanisms including sodium retention, blood volume expansion, abnormalities in renin-angiotensin-aldosterone-system (RAAS) and altered renal function with impact on sodium and fluid homeostasis [14–16]. Thus, salt sensitivity contains also the inability of the kidney to excrete excess sodium [17]. Yet, to our understanding, salt sensitivity has not been studied in the context of the renal ability to excrete sodium and water followed by a high salt load.

In our previously published study, haptoglobin (Hp) polymorphism-related salt sensitivity in sodium and water handling were reported [18]. In this study, we hypothesized that healthy individuals who display a low rate of sodium excretion after an acute high salt load react inappropriately with salt and water retention and are therefore called “salt-sensitive”. Understanding these individual differences based on the ability to excrete excess sodium after a high salt load and its effect on diuresis and other main urinary solutes such as urea could lead to individualized lifestyle interventions and recommendations for nocturia patients. Therefore, the aim of this study was to evaluate the effect of an acute salt load on diuresis, sodium, and urea excretion, in order to study the phenomenon of salt sensitivity.

## 2. Materials and Methods

### 2.1. Study Population

Twenty-one young, healthy male volunteers with median age of 26 years (21–27) and BMI 23 kg/m^2^ (20–24) participated in the study. The subjects were recruited through the university and the community via posters and flyers.

#### 2.1.1. Baseline Standardization and Exclusion Criteria

Young, healthy male volunteers were recruited to avoid any kind of interference regarding sodium and fluid homeostasis. Therefore, participants taking any type of medication or suffering from a chronic condition were excluded. All the trials were conducted after an overnight fasting period and a sober state in the morning to avoid the influence of diet and differences on hydration status. The subjects were not allowed to consume any alcoholic beverages for 24 h and were on their normal diet prior to each test day. All the subjects recorded a 3 day food diary including 2 week days and 1 weekend day, which used to evaluate their normal dietary intake.

### 2.2. Study Design

This is a prospective, interventional, pilot study conducted between June 2020 and December 2020. The study protocol was approved by the Ghent University Hospital Ethics Committee (EC 2019/0710; Clinical trial registration No : NCT04526340) and conducted in accordance with the Declaration of Helsinki. Written informed consent was obtained from all subjects.

#### 2.2.1. Experimental Protocol

**Figure 1:**
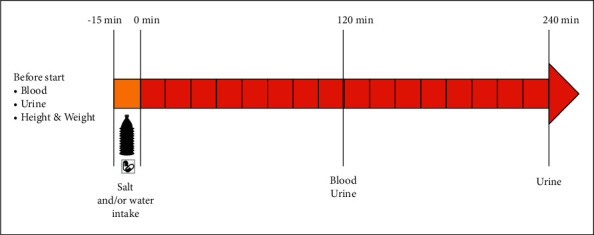
Diagram of experimental protocol.

In this study setting, each individual, acting as its own control, came to the hospital for three different tests on three different mornings. On the control day, the participants ingested 500 mL of still water over 15 minutes. On the 2 salt test days, the volunteers ingested either 8 or 12 salt capsules (1 g/capsule), along with 500 mL still water in 15 minutes (Figure 1).

In between testing days, there were at least two days to reduce interference from the previous high dose salt intake. The mineral composition of still water used (EVIAN RPET water bottle 500 mL) is presented in Table 1.

#### 2.2.2. Urine and Blood Collection

Upon arrival at the hospital, the volunteers delivered their first morning urine in a measuring cup and a urine sample was taken (baseline urine sample: U1). Afterwards, a 15 mL serum blood sample (B1) was collected. The height and the weight of the subjects were taken after the urine and blood samples were collected. Participants were asked to void again after 2 hours. Urine volume was registered and a second urine (U2) and a blood sample (B2) were taken. At the end of 4^th^ hour of the study period, the final urine sample (U3) was collected, after registration of the voiding volume.

### 2.3. Urine and Serum Analysis

Voided volumes, urine osmolality, and sodium, potassium, urea, and creatinine levels were analyzed from each urine sample. Osmolality was measured using freezing‐point depression (OSMO station OM‐6060, Arkray) [19]. Creatinine was measured using a compensated rate-blanked alkaline picrate method [20]. Urea was measured using an enzymatic assay (urease/glutamate dehydrogenase method) [21]. Sodium and potassium was measured using indirect potentiometry (Cobas 8000 modular analyzer, Roche) [22].

#### 2.3.1. Calculation of Renal Clearances

Serum osmolality was determined and utilized to calculate solute clearance (urine_osmolality_ × urine flow/plasma_osmolality_) and free water clearance (FWC) (urine flow−solute clearance).

#### 2.3.2. Calculation of Salt Sensitivity

Salt sensitivity was defined as alterations in sodium excretions due to high salt load. Therefore, to calculate the salt sensitivity of sodium excretion of each subjects after the high salt load, following equation was used:

(1) $$\begin{array}{c} \text{salt sensitivity}=\text{derivative}\left[\frac{\Delta \text{ sodium excretion}}{\Delta \text{ sodium intake}}\right],\\ \left(\text{a}\right)=\text{slope of the linear function}\left[\frac{\Delta y}{\Delta x}\right]. \end{array}$$

### 2.4. Salt Sensitivity Phenotyping

A secondary analysis to calculated salt sensitivity was carried out by dividing the study population into three groups/phenotypes on the basis of the derived value of rate of sodium excretion; <0: salt sensitive (SS), 0 to 0.99: moderate sensitive (SM), or ≥1: salt resistant (SR).

#### 2.4.1. Haptoglobin (Hp) Phenotyping and Plasma Hp Concentration

Starch gel electrophoresis was carried out for Haptoglobin phenotyping of haemoglobin-supplemented serum as described by Smithies et al. [23]. Hydrolysed starch (11.5%) was used to prepare the starch gel in a 0.1 mol/l Tris-citrate buffer (pH 8.86). Electrophoresis was performed for 1 h at 200 V in a 0.3 mol/l borate buffer (pH 8.4). Hp-Hb complexes were visualized using metal-enhanced peroxidase reagents.

Plasma Hp concentration was measured by fixed-time immuno-nephelometry using Dade Behring rabbit anti-human Hp polyclonal antibodies [24]. The Hp assay was standardized using CRM 470 reference material [25].

### 2.5. Calculation of Dietary Sodium Intake

Three day food diaries were used to calculate dietary sodium intake of each individuals using the sodium content of each food based on Belgian food composition database (Internubel.be).

### 2.6. Statistical Analysis

Clinical and biochemical parameters between the three test days were compared using the Wilcoxon signed‐rank test for two related samples. Within a test day, variance over 4 hrs was compared using the related-samples Friedman's Two-Way Analysis of variance, followed by a pairwise comparison with adjusted *p*-values by the Bonferroni correction for multiple tests. Differences in the salt sensitivity phenotype were compared using Kruskal–Wallis Test for K independent samples. Multiple regression analysis was used to determine the relationship between the total voided volume (ml/4 hrs), age (years), BMI (kg/m^2^), dietary sodium intake (g/day), salt sensitivity phenotype, Hp phenotype (Hp 1–1 vs. rest), Hp concentration (mmol/L), and urinary excretion of potassium, urea, and creatinine (mmol/4 hrs).

All continuous variables are reported as median (interquartile range). All analyses were performed using IBM SPSS Statistics for Windows, version 26 (IBM Corp., Armonk, NY., USA). A *p*-value < 0.05 was deemed statistically significant.

## 3. Results

### 3.1. Study Population

In the total 21 subjects participated in the study, 19 subjects completed all 3 test days. Two subjects voluntary withdrew after completing the control and 8 g salt day, due to discomfort caused by salt ingestion. Baseline characteristics are presented in Table 2. 42.9% of the population had ≤3.2 g dietary sodium intake ( = moderate, ≤ 8 g salt/per day [26]) per day. Only 14% of the study population met the WHO recommendation of <2 g dietary sodium/day. Hp phenotypic distribution of the study population comprised 38% Hp 1–1, 24% Hp 2–2 and 38% Hp 2–1. Body weight, serum osmolality, urine osmolality, or diuresis rate did not significantly differ between the trial days for each participant.

### 3.2. Effect of Salt and/or Water Ingestion on Fasted, Sober Healthy Young Men

#### 3.2.1. Before and after Effect

Control day: After the water load, increased diuresis rate and reduced urine osmolality were observed compared to baseline, and the effect was more prominent at 2 hrs (Table 3: A). However, no significant difference was observed in FWC or in urinary sodium/creatinine ratio at any time. On the other hand, compared to baseline, urinary potassium/creatinine ratio was significantly greater at all-time points, whereas urinary urea/creatinine ratio significantly increased at 2 hrs.

Salt days: After the salt load, compared to baseline, diuresis rate significantly increased for both salt dosages, but more profoundly for 12 g salt day at 4 hrs. However, urine osmolality showed no significant difference, whereas FWC reduced dramatically. Urinary sodium increased significantly, again more profoundly for 12 g of salt. A significant increase in urinary potassium and urea was observed only 4 hrs after ingestion of salt (Table 3: A).

#### 3.2.2. Salt Effect

Control vs salt days: Two hours after ingesting the salt, diuresis rate reduced significantly while increasing urinary osmolality. FWC reduced significantly and urinary sodium levels were significantly higher in the 12 g salt group, but not in the 8 g. In addition, ingestion of salt did not affect the urinary potassium or urea measured at 2 hrs (Table 3, B).

At 4 hrs, diuresis rate did not significantly differ between salt days and the control day. Nevertheless, increased urinary osmolality was observed in both salt groups, together with a significantly lower FWC. Urinary sodium and potassium were significantly higher in both salt trials than in the control, and similar to 2 hrs, ingestion of salt did not affect urinary urea at 4 hrs (Table 3, B).

#### 3.2.3. Dose Effect

**Figure 2:**
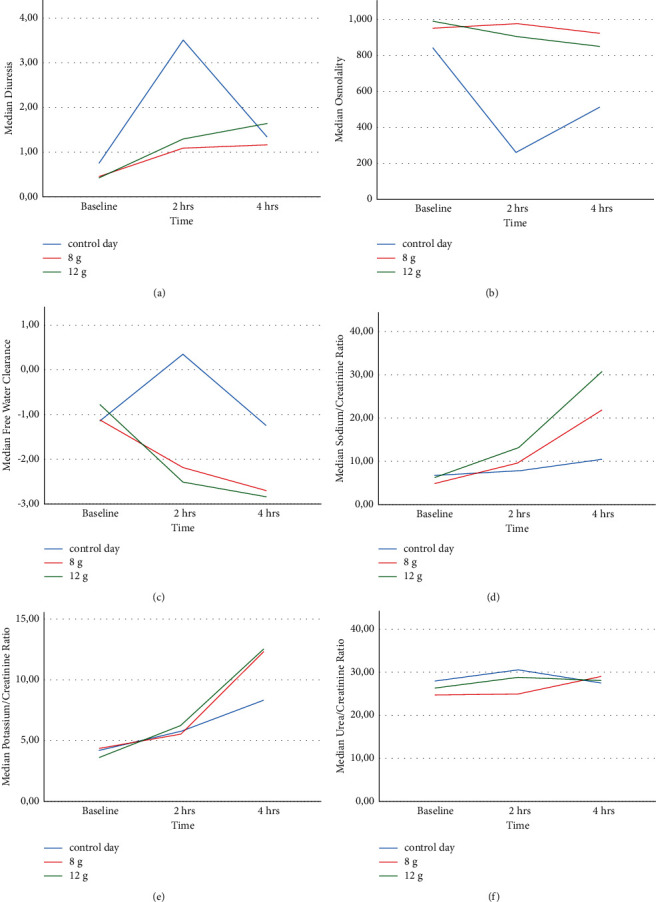
Trend of fluid balance for the 3 different test days. Diuresis rate ml/min (a), urine osmolality mOsm/kg (b), free water clearance ml/min (c), sodium/creatinine (mmol/mmol) (d), potassium/creatinine (mmol/mmol) (e), and urea/creatinine (mmol/mmol) (f).

8 g salt vs. 12 g salt: Compared with the 8 g of salt, 12 g of salt significantly increased diuresis and reduced urinary osmolality 4 hours after ingestion. 12 g of salt significantly increased urinary sodium at all times, but more intensely at 4 hrs (Table 3, B). No significant changes were observed in FWC or urinary potassium and urea levels between the two salt dosages.  Figure 2 shows the trends of fluid balance over the time on 3 different test days.

### 3.3. Relationship between Total Voided Volumes and Salt Sensitivity Phenotype

Comparison between the 3 salt sensitivity phenotypes showed that subjects with a higher rate of sodium excretion, further stated as salt sensitivity phenotype SM and SR, had significantly greater total voided volumes than SS subjects (SR: 480 and SM: 405 (320–450) vs. SS: 265 (197–300) ml/4 hrs, *p*=0.021*∗*). In multiple regression analysis, total voided volume produced after a high salt load (12 g), was positively associated with salt sensitivity phenotype, age, Hp phenotype, and creatinine excretion, whereas urea excretion was associated inversely with the total voided volume (Table 4).

## 4. Discussion

### 4.1. Summary of Evidence

The present study aimed to investigate the effect of an acute salt load on urine production and body fluid balance. One of the main findings of our study is that, in healthy young men, an acute high salt load (12 g) increased diuresis significantly compared to a moderate salt load (8 g), but the effect on diuresis was delayed by 4 hrs after ingestion. The total voided volume was significantly lower in the salt-sensitive phenotype, reflecting the subjects with a low rate of sodium excretion. Furthermore, a larger voided volume was associated with an increased rate of urinary sodium and creatinine excretion, age, Hp2-2/2–1 phenotypes, and decreased urea excretion. Therefore, our present study results suggest that an individual's ability to excrete excess urinary sodium is a main determinant of urine output following a high salt load.

The renal concentrating ability to excrete dietary sodium following a dietary salt load has been investigated earlier under different hydration and salt intake levels [27–29]. A study done by Rakova et al. reported an increased urinary osmolality and reduced FWC in healthy young men with a chronic high dietary salt intake (12 g/day) compared with those with a low dietary salt intake (6 g/day) [27]. Increased urinary sodium excretion was counterbalanced by a reduction in urea and potassium excretion. Even though no significant increase in urine volume was observed in the high salt intake compared to the low salt intake, urine volume was significantly higher in the highest urinary sodium excreting individuals compared to the lowest, which is in line with the results of our study. Similar results were reported in other studies [29, 30]. Another study that investigated the effect of varying hydration levels on urinary excretion of sodium after an acute salt load (5 g) reported an increase in sodium excretion and urinary flow rate in high hydration compared to low hydration status [28]. Excretion of urea increased in high hydration status and decreased in low hydration status, whereas potassium excretion remained unchanged [28]. Under the similar conditions of low hydration status, our study also showed a negative association between the total voided volume and urea excretion. The proposed mechanism behind this is an increase in vasopressin at low hydration level which limits the renal ability to excrete excess sodium selectively and rapidly, thus possibly causing a temporary sodium retention [28].

Beyond dietary intake, diurnal variations in urinary sodium excretion (low in early morning and high in afternoon to night hours) in free living individuals [31] as well as intra-individual variations (e.g., effect of rhythmic secretion of glucocorticoid and mineralocorticoid hormones [27], age, sex, genetic) have been discussed earlier [32]. In the present study, the impact of diurnal variability at the baseline was mainly controlled by having each study participant acting as his own control. The study was conducted under a highly controlled environment to minimize the impact as much as possible by recruiting only young, healthy men after an overnight fast and sober state.

In our previously published study, we demonstrated that Hp polymorphism-related salt sensitivity in Hp1-1 phenotype lacked circadian rhythm in diuresis, renal clearance of free water, and sodium, in contrast to Hp2-2/2-1 phenotypes [18]. Hp is a haemoglobin clearing plasma protein that displays three major phenotypes in humans as Hp 1-1, Hp 2-2, and Hp 2–1 [33], and Hp1‐1 has been associated in high BP and salt sensitivity [34]. Results of the current study suggest that subjects with Hp2-2/Hp2-1 phenotypes could excrete excess sodium efficiently by increasing urine volume after a salt load, possibly due to their salt resistant nature.

Therefore, major genetic and also baseline dietary intake differences between the different study populations may also play an important role in the renal ability to excrete sodium. For an example, a study done by Yatabe et al. reported that urinary sodium excretion reflected the changes in abrupt dietary salt intake at both 3 g/day and 20 g/day salt-diet in Japanese subjects (men = 5, female = 9, 21–26 years) [35], which was incompatible to the present study results. Japanese have a much higher average daily salt intake 9.9 g [36] than Belgians, who have an average daily salt intake of 5.8 g [37]. On the other hand, Japanese population has a strongly different Hp phenotype distribution than the Belgian population. In Japan, Hp1 allele frequency is about 0.25 (one of the lowest in the world), whereas the Belgian population has a Hp1 allele frequency of 0.40 [33]. This implies that salt sensitive Hp 1–1 subjects are very rare in Japan, whereas in Belgium they represent ±17% of the population [38]. Therefore, in Japanese population (with a low salt sensitivity), a steep sodium gradient is needed to observe differences (3 vs 20 g of salt as studied by Yatabe et al.), and this is not the case in Belgian population, which is much more salt-sensitive.

Taken together, the findings of these studies indicate the complex nature of renal sodium handling whilst regulating body water and electrolyte homeostasis in different populations and individuals.

### 4.2. Relevance and Implementation

Dietary salt restriction is widely recommended as a primary lifestyle intervention in the management of multiple conditions such as high BP, diabetes mellitus, and nocturia [10, 11]. In nocturnal polyuria (NP), one of the main causes of nocturia [39], an impaired circadian rhythm in renal handling of free water (vasopressin related) or sodium (renin-angiotensin-aldosterone-system (RAAS) and natriuretic peptides related) leads to excessive water and/or sodium diuresis at night [7, 40]. Dietary salt restriction has been reported to reduce nocturnal urine volume in renal allograft recipients with NP [12]. Thus, dietary salt restriction is a potential beneficial lifestyle modification for NP patients who lost circadian rhythm in renal handling of sodium and thus show enhanced sodium diuresis during nighttime [39]. However, the results of our current study suggest that general dietary modification recommendations such as salt reduction might not be beneficial for each patient. People with a low rate of sodium excretion or salt-sensitive individuals produce lower urine volumes as they may retain sodium along with water due to their impaired ability to excrete sodium. In contrast, subjects who are more salt-resistant may efficiently excrete excess sodium with a rapid increase in diuresis. Accordingly, understanding these individual variations in sodium handling is necessary for managing NP, with the emphasis on more personalized dietary recommendations, depending on the patients' salt-sensitivity profile.

Moreover, the European Association of Urology (EAU) recommendations for lifestyle interventions and type and amount of fluid intake in patients with voiding disorders needs further investigation. In this present study, we observed that pure water load induced fast diuresis, whereas water and salt load initially reduced diuresis and promoted late increase in diuresis. Taken together, electrolyte composition of drinks and time of consumption are other important aspects to consider when advising evening fluid intake in NP patients to promote prolonged fluid retention and to maintain fluid balance for an extended period. Likewise, age-related loss of circadian rhythm of renal sodium and water handling is the main cause of NP in the older population [7]. In conclusion, simple dietary modifications, regarding salt and fluid intake, would be beneficial for the overall health in the older population.

### 4.3. Study Limitations

Body sodium and fluid homeostasis are regulated by a complex, integrated neuro-hormonal system. However, in the present study, we did not measure BP and the effect on dipping patterns was not studied, which is an important limitation of this study. Furthermore, the results of the present analysis was limited to the acute effect of salt/water load over the following 4 hrs. However, with the current study design, it was too challenging to conduct the tests beyond 4 hrs, as the study participants were dehydrated and fasted for an extended period of time. In addition, the present study was a pilot study with relatively small sample size and conducted in young healthy men to avoid any kind interference regarding sodium and fluid homeostasis (e.g., chronic illness, hormonal shifts in women that could influence water handling) and in the morning to avoid any practical difficulties in baseline standardization. Thus, results may not be completely generalizable to other populations, particularly the elderly and chronically ill patients with impaired circadian rhythm, renal handling of water or sodium, or affected RAAS.

### 4.4. Future Research

Nevertheless, the results of this study provide important insights for well-designed future prospective studies. The primary focus of these future projects investigating the renal concentration ability to excrete excess sodium in NP patients should be building more refined individualized lifestyle recommendations. Therefore, future prospective studies with bigger sample size in a real life setting, without predefined fixed-time points for nighttime urine collection, together with frequency volume charts, BP measurements, the effect of dipping patterns, and hormonal analysis, are needed to further understand the role of low-, moderate-, and high-salt evening diets in NP patients in relation to salt sensitivity phenotypes.

## 5. Conclusions

In the present study, we demonstrated that in healthy young men, an acute high-salt load significantly increased diuresis compared to a moderate salt load, with a delay in diuresis effect by 4 hrs. Total voided volume was significantly lower in the salt-sensitive phenotype, also described as subjects with low rate of sodium excretion. Consistently, increased voided volume was significantly associated with salt resistance or less-salt-sensitive individuals, age, Hp2-2/2–1 phenotypes, increased urinary creatinine excretion, and decreased urea excretion. Dietary salt restriction is considered as a potential beneficial lifestyle modification for NP patients who lost circadian rhythm in urine production and therefore experience excessive urine production at nighttime. Circadian rhythm of urine production is regulated by the circadian clocks present in the brain, kidney, and bladder. In vivo, an acute high salt load could alter the clock time and drive an unscheduled homeostatic response. The findings of the present study reveal important considerations for future prospective trials which aim to build effective, personalized lifestyle recommendations for NP patients based on salt-sensitivity phenotypes.

## Figures and Tables

**Table 1:** Mineral composition of water obtained from the water bottle label.

	Sodium	Potassium	Calcium	Magnesium	Nitrates	Bicarbonates	Sulfates	Silica	Chlorides
Unit mg/L	6.5	1	80	26	3.8	360	14	15	10

**Table 2:** Baseline demographics of the population.

Parameter	Results (*n* = 21)			
Age (years)	26 (21–27)			
BMI (kg/m^2^)	23.6 (20.5–24.7)			
Dietary sodium intake (g/day)	3.89 (2.51–4.79)			
Moderate intake ≤ 3.2 g/day (%)	9 (42.9)			
High intake > 3.2 g/day (%)	12 (57.1)			
Haptoglobin phenotype (%)				
1 Hp 1–1	8 (38)			
2 Hp 2–1	8 (38)			
3 Hp 2–2	5 (24)			
Salt sensitivity phenotype (%)				
1 SS	6 (31.6)			
2 SM	12 (63.1)			
3 SR	1 (5.3)			
	**Day 1 (n**–**21)**	**Day 2 (n** **=** **21)**	**Day 3 (n** **=** **19)**	**P value**
Body weight (kg)	75 (68.3–80.5)	74 (68.7–80.5)	73.3 (68–79)	NS
**Plasma**				
Osmolality (mOsm/Kg)	287 (285–290)	288 (288–291)	288 (286–291)	NS
Sodium/Creatinine (mmol/mmol)	153 (138–170)	152 (146–160)	150 (141–159)	NS
Potassium/Creatinine (mmol/mmol)	4.5 (4–5)	4.3 (4.1–4.6)	4.4 (4–4.5)	NS
Urea/Creatinine (mmol/mmol)	35 (29–44)	35 (33–43)	35 (29–42)	NS
**Urine**				
Diuresis rate (mL/min)	0.8 (0.4–0.9)	0.5 (0.3–0.8)	0.4 (0.3–0.6)	NS
Osmolality mOsm/kg	842 (713–1001)	951 (840–1039)	988 (596–1049)	NS
Sodium/Creatinine(mmol/mmol)	6.8 (5.4–13.4)	5.2 (3.6–7.8)	6.2 (5.7–7.3)	NS
Potassium/Creatinine(mmol/mmol)	4 (2–5.6)	3.9 (2.5–4.8)	3 (1.9–7)	NS
Urea/Creatinine (mmol/mmol)	27 (22–31)	26 (20–29)	24 (20–32)	NS

*∗p* < 0.05 for control vs. 8 g salt, †*p* < 0.05 for control vs. 12 g salt and Δ*p* < 0.05 for 8 g vs. 12 g salt; Wilcoxon signed rank test. BMI = body mass index; Hp = haptoglobin phenotype; SS = salt sensitive; SM = moderate sensitive; SR = salt resistant; NS = not significant. This study was conducted in 3 different test days as day 1, 2, and 3. Therefore, clinical parameters of plasma and urine samples taken at the baseline/before the test started on different test days (as day 1, 2, and 3) are shown in the bold values.

**Table 3:** Time course of changes in diuresis rate, urine osmolality, FWC, and solute/creatinine ratios within the test days (A) and between the test days (B), over 4 hrs after salt and/or water ingestion.

(A) Within the test days				
	Control	8 g salt	12 g salt	

Diuresis (mL/min)				
2 hrs	3.5 (1.4–4.3)	1.1 (0.8–1.5)	1.2 (0.8–1.8)	
4 hrs	1.3 (0.8–2.0)	1.2 (0.8–1.5)	1.6 (1.0–1.9)	
* P* value	Baseline vs. 2 hrs: < 0.001a	Baseline vs. 2 hrs: 0.006a	Baseline vs. 2 hrs: 0.006a	
	Baseline vs. 4 hrs: 0.026a	Baseline vs. 4 hrs: 0.021a	Baseline vs. 4 hrs: 0.001a	
Urine osmolality (mOsm/kg)				
2 hrs	260 (166–910)	927 (824–1072)	905 (771–1056)	
4 hrs	517 (384–742)	900 (796–982)	849 (764–960)	
* P* value	< 0.001^∗∗^	NS	NS	
Free water clearance (mL/min)				
2 hrs	0.3 (−2.6–1.4)	−2.2 (−3.0–(−1.5))	−2.5 (−3.3–(−1.8))	
4 hrs	−1.2 (−1.7–(−0.6))	−2.7 (−2.9–(−1.5))	−2.8 (−3.5–(−1.9))	
* P* value	NS	Baseline vs. 2 hrs: 0.006a	Baseline vs. 2 hrs: <0.001a	
		Baseline vs. 4 hrs: 0.021a	Baseline vs. 4 hrs: 0.002a	
Sodium/creatinine (mmol/mmol)				
2 hrs	7.8 (6.3–12.7)	12.4 (5.8–16.9)	13.1 (11.6–20.5)	
4 hrs	10.2 (6.5–16)	23.6 (16.2–31.4)	30.6 (26.3–38.6)	
* P* value	NS	< 0.001^∗∗^	< 0.001^∗∗^	
Potassium/creatinine (mmol/mmol)				
2 hrs	5.7 (3.9–8.5)	5.8 (4.1–7.8)	6.2 (3.5–7.7)	
4 hrs	9.4 (6.2–11)	13 (9.7–15.1)	12.5 (9.3–17.7)	
* P* value	Baseline vs. 2 hrs: 0.001a	Baseline vs. 4 hrs: <0.001a	Baseline vs. 4 hrs: <0.001a	
	Baseline vs. 4 hrs: <0.001a	2 hrs vs. 4 hrs: 0.003a	2 hrs vs. 4 hrs: 0.004a	
Urea/creatinine (mmol/mmol)				
2 hrs	30 (25–32)	25 (21–32)	28 (20–34)	
4 hrs	28 (23–33)	29 (22–36)	28 (24–35)	
* P* value	Baseline vs. 2 hrs: 0.005a	Baseline vs. 4 hrs: 0.018a	Baseline vs. 4 hrs: 0.024a	
*(B) Between the test days*				
	Control	8 g salt	12 g salt	*P* value

Diuresis (mL/min)				
2 hrs	3.5 (1.4–4.3)	1.1 (0.8–1.5)	1.2 (0.8–1.8)	0.001^∗^ 0.002†
4 hrs	1.3 (0.8–2.0)	1.2 (0.8–1.5)	1.6 (1.0–1.9)	0.010∆
Urine osmolality (mOsm/kg)				
2 hrs	260 (166–910)	927 (824–1072)	905 (771–1056)	< 0.001^∗^ < 0.001†
4 hrs	517 (384–742)	900 (796–982)	849 (764–960)	0.001^∗^ 0.003† 0.026∆
Free water clearance (mL/min)				
2 hrs	0.3 (−2.6–1.4)	−2.2 (−3.0–(−1.5))	−2.5 (−3.3–(−1.8))	0.002^∗^ 0.002†
4 hrs	−1.2 (−1.7–(−0.6))	−2.7 (−2.9–(−1.5))	−2.8 (−3.5–(−1.9))	0.001^∗^ 0.013†
Sodium/creatinine (mmol/mmol)				
2 hrs	7.8 (6.3–12.7)	12.4 (5.8–16.9)	13.1 (11.6–20.5)	0.006† 0.027∆
4 hrs	10.2 (6.5–16)	23.6 (16.2–31.4)	30.6 (26.3–38.6)	0.002^∗^ < 0.001† 0.006∆
Potassium/creatinine (mmol/mmol)				
2 hrs	5.7 (3.9–8.5)	5.8 (4.1–7.8)	6.2 (3.5–7.7)	NS
4 hrs	9.4 (6.2–11)	13 (9.7–15.1)	12.5 (9.3–17.7)	< 0.001^∗^ 0.007†
Urea/creatinine (mmol/mmol)				
2 hrs	30 (25–32)	25 (21–32)	28 (20–34)	NS
4 hrs	28 (23–33)	29 (22–36)	28 (24–35)	NS

^∗∗^ *p* < 0.05 for related-samples Friedman's Two-Way Analysis of variance and ^a^*p* < 0.005 for pairwise comparison with adjusted *p* values by the Bonferroni correction for multiple tests. NS = not significant. *∗p* < 0.05 for control vs. 8 g salt, †*p* < 0.05 for control vs. 12 g salt and Δ*p* < 0.05 for 8 g vs. 12 g salt; Wilcoxon signed rank test. NS = not significant.

**Table 4:** Multiple Regression analysis of total voided volume/4 hrs.

Independent variables	ß	t	P value
Age (years)	0.464	3.057	**0.014** ^∗^
BMI (kg/m^2^)	−0.359	−1.464	0.177
Dietary sodium intake (g/day)	−0.006	−0.030	0.976
Hp concentration (mmol/L)	0.540	1.931	0.086
Urinary potassium excretion (mmol/4 hrs)	0.029	0.117	0.909
Urinary urea excretion (mmo/4 hrs)	−0.688	−2.716	0.024^∗^
Urinary creatinine excretion (mmol/4 hrs)	0.872	2.321	0.045^∗^
Hp phenotype Hp1-1 [1] vs. rest [2]	0.673	2.744	0.023^∗^
Salt sensitivity phenotype SS [1], SM [2] vs. SR [3]	0.654	3.582	0.006^∗^

Dependent variable: total voided volume/4 hrs. Data are expressed as standardized regression coefficients (ß), *t*-value and *P* values. *∗p* < 0.05. NS = not significant. The bold value is the significant *p* value for age.

## Data Availability

The data used to support the findings of this study are available from the corresponding author upon request.
